# Failing to attend versus failing to stop: Single-trial decomposition of action-stopping in the stop signal task

**DOI:** 10.3758/s13428-022-02008-x

**Published:** 2022-11-07

**Authors:** Ricci Hannah, Vignesh Muralidharan, Adam R Aron

**Affiliations:** 1 Department of Psychology, University of California San Diego, La Jolla, CA USA; 2 Centre for Human & Applied Physiological Sciences, King’s College London, London, UK

**Keywords:** Inhibitory control, Attention, Impulsivity, Reaching movement, Online, Human

## Abstract

The capacity to stop impending or ongoing actions contributes to executive control over behavior. Action-stopping, however, is difficult to directly quantify. It is therefore assayed via computational modeling of behavior in the stop signal task to estimate the latency of stopping (stop signal reaction time, SSRT) and, more recently, the reliability of stopping in terms of the distribution of SSRTs (standard deviation, SD-SSRT) and the frequency with which one outright fails to react to a stop signal (trigger failures, TF). Critically, the validity of computational estimates remains unknown because we currently have no direct readouts of behavior against which to compare them. Here, we developed a method for providing single-trial behavioral readouts of SSRT and trigger failures. The method relies on an adaptation of the stop signal task in which participants respond by moving a computer mouse. In two online experiments, we used movement kinematics to quantify stopping performance (SSRT, SD-SSRT, and TF), and then applied the standard Race Model and recent BEESTS model in order to examine the convergent validity of the methods. Overall, we demonstrate good correspondence between kinematics- and model-based estimates of stopping performance at the group and individual level. We conclude that the new method provides valid estimates of stopping performance that, unlike model-based estimates, can be read out at the level of single trials. Our approach might therefore be useful for interrogating single-trial neurophysiological correlates of stopping and for large-scale, online studies of behavioral stopping.

## Introduction

Action-stopping is an important aspect of executive control, helping to ensure we behave appropriately in a given situation. For example, resisting the urge to swear when angry or reach for a doughnut when dieting. Stopping is typically studied with the stop signal task, in which participants prepare motor responses but are sometimes then cued to stop the impending response. Since successful stopping results in the omission of a response, behavioral stopping cannot be directly observed or quantified. Instead, stopping research relies on a computational model, the Race Model (Logan & Cowan, 1984), to estimate the stop signal reaction time (SSRT) as a marker of stopping efficacy. The Race Model has been widely adopted throughout psychology and neuropsychiatry as a model of action-stopping and executive control [for reviews, see (Aron, 2011; Aron & Poldrack, 2005; Bari & Robbins, 2013; Chambers, Garavan, & Bellgrove, 2009)]. It helped lay the groundwork for systematic investigation into the neuroanatomy of stopping and, in turn, motivated a prefrontal-basal-ganglia-thalamocortical network model of stopping [as reviewed in (Hannah & Aron, 2021)].

Recently, a new computational model of action-stopping, relying on Bayesian parameter estimation, was developed (Matzke et al., 2013; Matzke, Love, & Heathcote, 2017). The Bayesian Estimation of Ex-Gaussian STop-Signal (BEESTS) reaction time distributions model offers to extend on the Race Model and provide a richer description of stopping behavior by allowing one to quantify not just its latency, but also its reliability (Matzke et al., 2013, 2017). Whereas the main output from the Race Model is a single estimate of SSRT per person, BEESTS additionally estimates the intra-individual standard deviation of SSRT (SD-SSRT) as an indicator of the variability of stopping latencies. It also provides an estimate of so-called trigger failures (TF), instances where a failure to stop presumably results from an attentional lapse and associated failure to trigger the stop process. Finally, BEESTS is able to account for any potential bias introduced into the calculation of SSRT by the presence of trigger failures.

The BEESTS model is therefore a potential boon to the study of individual differences in executive control because it acknowledges, as has been pointed out recently, that the average ‘speed’ of stopping alone is unlikely to fully account for the success or failure of control (Hannah & Aron, 2021). It also potentially allows one to identify the specific processing stage at which inter-group differences or intra-individual changes arise (e.g., attentional versus implementational). For example, some work has indicated that deficits in action-stopping in individuals with attention deficit hyperactivity disorder may have more to do with impaired attentional processes that lend to issues in selecting and triggering the stop process, than with implementing the stop process itself (Weigard et al., 2019).

A potential criticism of BEESTS, however, is that the validity of the stopping performance estimates that it produces is unclear because direct readouts of stopping latency and trigger failures, against which they can be compared, have so far remained elusive. Incidentally, the same applies to Race Model estimates of stopping latency and the problem is exemplified by the fact that sometimes the BEESTS model produces estimates of SSRT that are considerably (> 40%) shorter than Race Model estimates (Skippen et al., 2019).

Our primary aim was to address this issue by developing a method for providing single-trial behavioral readouts of stopping latency and trigger failures. Our approach relied on an adaptation of the stop signal task that required participants to make responses by moving a computer mouse. The benefit of this approach over the typical one, where participants respond via key presses such that the outcome is binary (Bissett et al., 2021; Jana et al., 2020; Skippen et al., 2019; Weigard et al., 2019), is that the mouse movements provide a continuous readout of actions as they unfold, from their initiation through to their completion, and thus allow one to directly observe if and when actions are interrupted. That is, we expected that the movement kinematics would carry information about the stop process.

Although some previous studies have used continuous readouts of movement kinetics/kinematics (Atsma et al., 2018; Brunamonti et al., 2012; de Jong et al., 1990; Morein-Zamir et al., 2006; Venkataramani et al., 2018) and muscle activity (Atsma et al., 2018; Goonetilleke et al., 2010, 2012; Hannah et al., 2020; Jana et al., 2020; McGarry & Franks, 1997; Raud & Huster, 2017) to examine the latency and variability of stopping, none considered the potential to quantify trigger failures. Additionally, most of the methods relied on specialized laboratory equipment. Thus, a secondary aim was to develop a simple and inexpensive method that is suitable for online studies, which in turn offers a way to address challenges to the reproducibility and generalizability of psychological research by enabling the study of large and demographically diverse samples and facilitating replication of experiments.

In two online behavioral experiments, we quantified stopping performance (SSRT, the standard deviation of SSRT and trigger failures) using movement kinematics, the Race Model and the BEESTS model in order to examine the convergent validity of the methods.

## Methods

### Participants

All participants were healthy, adult humans who provided informed consent and were compensated $6.50/hour. They were recruited via an online participant database (https://Prolific.co) and completed the experiments online. The experiments were approved by the UCSD Institutional Review Board.

#### Experiment 1

No previous study has directly contrasted kinematic- and model-based estimates of stopping performance. Therefore, for the first experiment, we chose a 25-ms difference between estimates of SSRT as the minimum meaningful difference of interest [i.e. ~10 % difference in the means assuming an average SSRT of ~250 ± 40 ms (Aron et al., 2007; Hannah et al., 2020; Jana et al., 2020; Skippen et al., 2019; Smittenaar et al., 2013; van den Wildenberg et al., 2009; Weigard et al., 2019)], and a correlation of *r* = 0.5 as the minimum meaningful relationship of interest between the estimates of SSRT. We estimated that a sample size of ≥ 23 (difference score) and ≥ 26 (correlation) would be required to detect such effects with an alpha of 0.05 and power of 0.8. We therefore decided to recruit 40 participants, assuming an attrition rate of 25% after filtering data based on performance criteria (see below), to leave us with a final sample size of ~30. Note that in some cases we were predicting a null result (i.e., no difference between estimates of SSRT across methods), therefore we also computed Bayes factors to enable interpretation of null results, i.e., the strength of evidence for the null hypothesis (see *Statistical Analyses*).

Forty participants completed the experiment (mean age 35 ± 10 years, 23 males, all right-handed). Data from 17 participants were excluded due to their behavior in the stop signal task not meeting performance criteria (e.g., failed stop response times exceeded go response times, probability of successful stopping (pStop) was less than 25% or greater than 75%, rate of go errors exceeded 20%; Table 1). The remaining sample consisted of 23 participants (mean age 35 ± 10 years, 14 males). The rate of exclusions (~40%) was broadly similar to that in another online version of the standard stop signal task using similar criteria [~30% (Jana & Aron, 2021)].

**Table 1:** Number of people from total sample meeting specific performance criteria

		Exp. 1 (*n* = 40)	Exp. 2 (*n* = 52)
FSRT<GoRT (*n*)	Home	34	38
	Target	38	49
pStop 25–75% (*n*)	Home	29	40
	Target	29	50
Go Errors < 20% (*n*)		34	50
All criteria met (*n*)		23	34

Key: *FSRT* failed stop reaction time, *GoRT* go reaction time, *pStop* probability of stopping

#### Experiment 2: Replication study

Here we wished to replicate the results of the first experiment. We planned to recruit a final sample of at least 30 people, and therefore recruited a sample of 52 participants (mean age 24 ± 7 years, 39 males, all right-handed) with an assumed attrition rate of 40% based on experiment 1. After removal of data from 18 participants, due to poor task performance (see above; Table 1) or for suspected use of a mouse track pad rather than an external mouse (see *Experimental setup*), the final sample consisted of 34 participants (mean age 24 ± 7 years, 26 males).

### Experimental setup

**Fig. 1:**
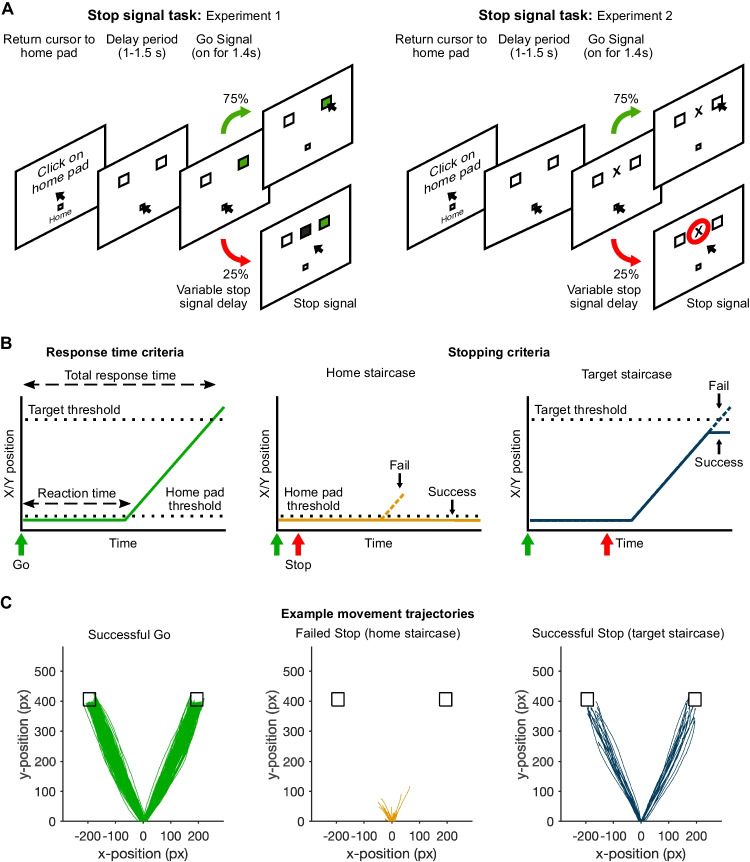
**a** Stop signal task in experiments 1 and 2. The go and stop cues differed across the two experiments, but otherwise the task was the same. Clicking on the home pad resulted in the presentation of two potential targets (*white squares*). After a delay period lasting 1–1.5 s, the go signal was presented for 1.4 s, which was the amount of time participants were allowed to make a response. In experiment 1, the go signals involved one of the target squares changing color from white to green. In stop trials, a stop signal appeared at a variable delay (stop signal delay, SSD), and in experiment 1 the stop signal was a black square that appeared between the two targets. In experiment 2, we changed the go signal so that the appropriate target was indicated by a letter appearing between the two targets, ‘T’ indicated left and ‘X’ indicated right. The purpose was to slow down response times in the primary go task to minimize the chance of the stop signal delay hitting a floor (i.e., the minimum SSD of 33.3 ms). In experiment 2, the stop signal was changed to a red circle, to accommodate the new go signal. **b** Schematic of response time criteria for go trials and stopping criteria for the home and target staircases on stop trials. Reaction time reflects the time between the go signal and the cursor leaving the home pad. Total response time reflects the time between the go signal and the cursor entering the target. Two different definitions of stop success and failure were used to adjust two different stop signal delay tracking staircases. For the home staircase, a stop was deemed successful if the cursor remained within the home pad, whereas for the target staircase, stopping was successful if the cursor did not enter the target. **c** Exemplar movement trajectories across different trial types for a participant in experiment 1. *Black squares* represent the left and right targets. *Colored lines* represent traces from individual trials shown from the time the cursor left the home pad to when the cursor entered the respective target (Go trials), and to the time of the maximum resultant XY displacement (i.e., when the movement came to a halt; failed stop and successful stop trials). *Axes* represent the position of the cursor with respect the center of the home pad in the *x*-plane and the top of the home pad in the *y*-plane. Units are pixels. Note that the responses could be stopped at stage right up to the point of reaching the target (see target staircase), which illustrates the lack of a ballistic stage in responding (de Jong et al., 1990; McGarry & Franks, 1997)

Instructions provided to participants recommended that they sit approximately 60 cm or arms’ length from the computer monitor, with the monitor approximately at eye level. Participants made motor responses by moving a computer mouse from a ‘home pad’ to targets on the monitor (Fig. 1a). The size and location of the home pad and targets were set as proportions of the browser window dimensions (Table 2), 1% window height and 2.5% window width, respectively. Therefore, the absolute size and position of targets varied for different participants as a function of the different monitor resolution and window dimensions. Stimulus timings were presented as a function of screen refresh rate (Table 2).

**Table 2:** Operating systems, browsers, and monitor characteristics used by individuals included in the final sample

		Exp. 1 (*n* = 23)	Exp. 2 (*n* = 34)
Operating system (*n*)	Windows	7	34
	Mac	15	0
	Other	1	0
Browser (*n*)	Chrome	17	27
	Firefox	3	7
	Other	3	0
Screen refresh rate (Hz)		60 ± 19	76 ± 42
Window width (px)		1627 ± 325	1630 ± 252
Window height (px)		834 ± 173	803 ± 141

Participants were asked to use an external computer mouse rather than a track pad and to reduce the cursor sensitivity as low as possible. The rationale for this was to encourage larger and longer duration movements than are usual, as we expected these to be more amenable to stopping than the smaller/briefer (i.e., ballistic) movements. The target size was also chosen to maintain an appropriate balance between accuracy and speed of the movements, i.e., it was not so large that movements could be performed ballistically with little concern for direction/accuracy.

Since we could not explicitly verify that people followed the instructions to use an external mouse, we included a brief reaction time task as the start of the experiment, which served to both familiarize participants with the task and allowed us to screen for individuals whose movement durations (total response time minus the reaction time; see Fig. 1) were too short according to our pilot work (< 200 ms). Participants were discouraged from participating further if their responses were consistently below this criterion. We also visually inspected the raw position-time data traces during analysis to screen for movement trajectories consistent with the use of a mouse track pad. We looked for highly curved movements that might occur with simple movements of the wrist, and ‘two-step’ movements that might occur because the reduced sensitivity of the mouse would not permit the cursor to be moved to the target in a single movement. Two participants displayed such movements and their data were excluded.

### Stop signal task

The task was coded using the JavaScript library, jsPsych (de Leeuw, 2015), and the jsPsych plugin, jsPsych-psychophysics (Kuroki, 2021), and run on the participants’ own computers using a web browser (Table 2). Responses in the task were made by moving a computer mouse cursor to a target on the screen. Each trial of the task began with participants moving the cursor to, and clicking on, a small square labelled the ‘home pad’ (Fig. 1). This initiated the presentation of two, white target squares on the screen. The targets were presented at an eccentricity of 25° with respect to the home pad. After a brief delay, the go signal was presented, cueing participants to move the mouse cursor to the target. Participants were encouraged to reach the target in a single, smooth movement, and to be as fast and as accurate as possible (Go trials). If participants did not reach the target within the given time period, the trial timed-out and a message saying ‘Too slow’ was presented. On 25% trials, a stop signal was presented after a variable SSD. Participants were told to stop their movements as soon as possible. The SSD was adjusted based on the success of stopping, where it increased and decreased by ~33.3 ms (rounded to the nearest frame) after successful and failed stops, respectively. Note that whilst this SSD step was suitable for monitor refresh rates of 60, 120, and 240 Hz, some monitors had refresh rates that were not perfectly divisible by the SSD step (5/23 in experiment 1 and 9/34 in experiment 2). Whilst this would have affected the absolute step size and thus the efficiency of the tracking procedure, it did not affect the overall effectiveness of the tracking procedure because stopping staircases converged regardless of the monitor refresh rates. Data here are all presented as the actual time of stimuli, rather than the time intended for their presentation during the experiment.

A key difference between this version and the standard version of the stop signal task is that we adopted two separate definitions of response and two definitions of stop success within the same task (Fig. 1b). In one instance, we defined a response as any movement causing the mouse to exit the home pad, which was only a few pixels across (8 ± 2 and 8 ± 1 pixel in experiments 1 and 2), and in the other a response was defined as when the cursor entered the target within the specified time window. These two definitions allowed us to use two separate SSD staircases, run independently of one another, to track the success of stopping movements in the planning and execution phases of movement. In one case, stopping was considered successful if the cursor remained within the home pad (home staircase), and in the other, stopping was considered successful if the movement was stopped before the cursor entered the target (target staircase). Importantly, participants were unaware of the two different stop criteria, and were only told that they should stop as soon as possible whenever they saw a stop signal. There was no upper limit to the SSD in either staircase.

This overall set-up conveyed two important benefits. The first is that we expected it would allow us to evaluate stopping latencies during both the planning and execution phases of movement (i.e., interrupted prior to or subsequent to movement initiation), and confirm that they rely on similar principles and processes. This would then provide the basis for using movement kinematics during the stopping of ongoing movements to infer the latency of stopping on a trial-by-trial basis. The second benefit of our approach is that we predicted we could directly observe trigger failures at the single-trial level. The following paragraphs explain our rationale.

We first assumed that stopping performance in our reaching version of the task relied on similar processes as standard versions of the task, and hence that behavior could be readily explained by the Race Model [for similar applications of the Race Model to the stopping of reaching/pointing movements, see (Atsma et al., 2018; Brunamonti et al., 2012; Mirabella et al., 2009; Venkataramani et al., 2018)]. In other words, two independent processes, a go and a stop, race to completion. The race is usually thought to occur during the planning stage, so that the outcome determines whether or not movement is generated, which in most studies is whether a button is pressed or not. However, this discrete categorization is misleading as movements can often be interrupted or stopped at any time prior to or following their initiation (de Jong et al., 1990; Georgopoulos et al., 1981). This is true even of ballistic button presses, where successful stops are sometimes accompanied by bursts of agonist muscle activity that are initiated but cancelled before a response is registered (Atsma et al., 2018; Hannah et al., 2020; Jana et al., 2020; Raud & Huster, 2017). By examining entire movement trajectories and employing separate staircases, we can separately interrogate stopping in each phase of movement: home staircase (planned/uninitiated movement) and target staircase (initiated/ongoing movement). The main difference, as far as the Race Model is concerned, is that for the stopping in the execution phase, the race continues after movement has been initiated to a new threshold reflecting the completion of the movement (de Jong et al., 1990; Venkataramani et al., 2018).

We then reasoned that evaluating the movement trajectories for those trials in which movement stopped short of the target would allow us to infer the completion of the stop process as the time at which the movement was interrupted/stopped. This time minus the stop signal delay could then be considered a direct measure of stopping latency on a given trial. For the target staircase, the logic is straightforward and we can measure the time at which the movement came to a halt in successful stop trials. However, successful stops for the home staircase will exhibit little-to-no movement. Nevertheless, we predicted that even when a movement is initiated, and the trial is effectively a failed stop, participants would still attempt to stop the movement because they were unaware of the home staircase criterion. This is because they were not explicitly told about the criterion and did not receive explicit trial-wise feedback about stop performance that otherwise might have informed them. Moreover, the combination of a long movement duration (~400 ms) and short SSDs should mean that participants nearly always have enough time to stop an initiated movement before it reaches the target (the criterion for a successful stop in the target staircase). The implication is that we can measure the single-trial latency of stopping of movements soon after their initiation (home staircase, failed stop trials) or later on and closer to completion (target staircase, successful stop trials).

Finally, we predicted that there will occasionally be failed stop trials for the home staircase where the participant does not stop the ongoing movement and does indeed reach the target, despite the ample time available to stop. The assumption here is that the stop process was simply not triggered, i.e., there was a trigger failure.

### Procedure

Participants first completed two blocks of 32 trials of a choice reaction time task, which formed the basis of the stop signal task. The reaction time task was followed by two blocks of 32 trials where participants practiced the stop signal task. This familiarized participants with the task and also helped titrate the SSD so that it was at an appropriate level at the start of the main experiment. Participants completed 18 blocks of 32 trials of the stop signal task in the main part of the experiment. Each block of the stop signal task contained four stop trials for the home staircase and four stop trials for the target staircase, divided evenly between left and right targets. At the end of each block, participants received feedback about their average total response time, and were encouraged to speed up if their responses began to slow down or to do their best to stop if stopping accuracy was <30 or >70%. Participants were allowed to rest as long as they liked between blocks.

### Data recording

Cursor movements, i.e., times and positions in the *x*- (left-right) and *y*-planes (up-down), were recorded from onset of cursor movement at a recording frequency equivalent to the screen refresh rate (Table 2). Screen and window dimensions, browser and operating system were all automatically detected and recorded. All data were saved on server for later analysis.

### Data analyses

#### Response times and errors

The time between the go signal and the cursor exiting the home pad was considered the reaction time, and the time between the go signal and the cursor entering the target was considered the total response time (Fig. 1). Response errors could come in three forms: response omission, choice errors (hitting the wrong target) and missed targets (aiming for, but missing, the correct target). The number of response errors on go trials was expressed as a percentage of the total number of go trials.

#### Stopping latencies

Stopping latencies were estimated using three different methods, each used twice to estimate the latency for the home and target staircases. First, we applied the Race Model and used the integration method to estimate SSRT_RM_ (Verbruggen et al., 2019). The Race Model is the standard way of assessing stopping and has been applied in hundreds of studies. If our data did indeed conform to various predictions of the model, then the resulting SSRT_RM_ values should provide a reasonable starting point for evaluating the convergent validity of our kinematic estimates. Two estimates of SSRT_RM_ were produced by using stopping performance (probability of stopping and SSDs) along with the reaction time distribution for the home staircase (SSRT_RM-H_) and the total response time distribution for the target staircase (SSRT_RM-T_).

**Fig. 2:**
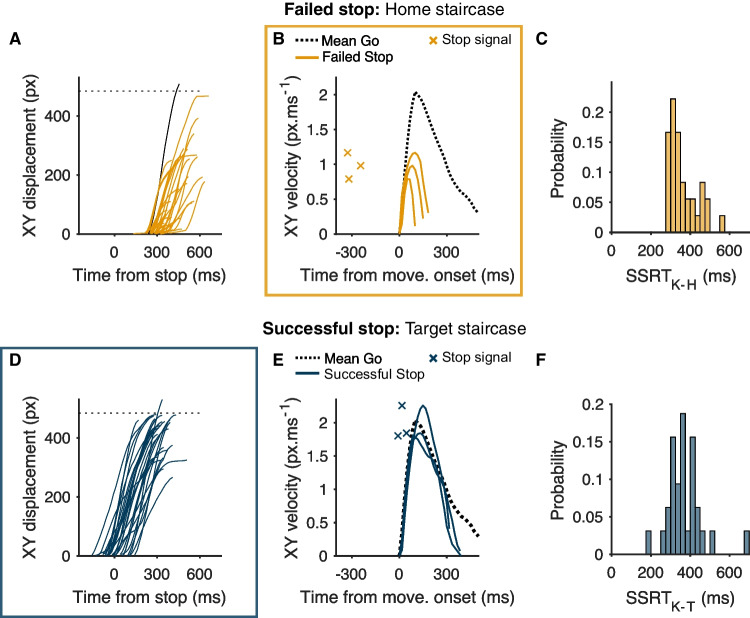
Exemplar data from a single subject. **a**, **d** Resultant displacement of the cursor (in pixels), with each trace representing a single trial. *Dashed horizontal line* reflects a pseudo-threshold for a movement being registered as entering the target and is shown for illustrative purposes only, since in reality the target was square and could be entered at various points. Although trials in the home staircase are labeled failed stops, movements were generally still cancelled before the target was reached. The *black trace* here represents a trial where the individual failed to stop before reaching the target. This trial is considered a trigger failure (TF_K-H_). In **d**, one trace appears to exceed the pseudo-threshold for a response but, in reality, the cursor did not enter the target. **b**, **e** The resultant velocity of the cursor for select stopping trials, as well as the average across all go trials. Velocity profiles in failed stop trials (**b**) have a smaller peak and decline much more rapidly compared to go trials. We took the time of the peak relative to the time of the stop signal as a single-trial measure of stopping latency. In **e**, the stop signals arrive much closer to movement onset and so the velocity profiles of successful stops are very similar to those of go trials for much of their time course, diverging only later when the movement is already decelerating. The time of the peak velocity is less informative about the stop process here, and so we simply used the time at which the cursor reached its maximum displacement (**d**) as the completion of the stop process and took this time relative to the stop signal as the latency of stopping. **c**, **e** The distribution of stopping latencies across trials (SSRT_K_) measured using the velocity and displacement methods

Secondly, we used the cursor movement kinematics to measure SSRT. Point-to-point movements typically display a bell-shaped velocity profile (Atkeson & Hollerbach, 1985; Kelso et al., 1979). We predicted that the velocity–time profiles for stopped movements of the home staircase would exhibit smaller and earlier peaks compared to go trials, reflecting their interrupted nature (see Fig. 2b). Moreover, we expected that the timing of the peak (i.e., the point at which the velocity starts to decline) on those trials reflected the onset of the stop process. For each trial, we first calculated the resultant displacement of the cursor at each time during the movement relative to the point at which it left the home pad. We then used the central difference method to estimate the resultant velocity at each time. Finally, we measured the time of the peak velocity relative to that of the stop signal as a kinematically derived estimate of SSRT (SSRT_K_). The same method could not be used for the target staircase because the stop signal arrives so late that the stop process will only start to impact the velocity profile after the natural peak of an uninterrupted movement, when movement velocity is already declining (see Fig. 2b). We also observed substantial heterogeneity in the shape of the velocity-time profiles across trials at this stage in the movement, which made the single-trial measurement of stopping latencies a challenge. We therefore used a simpler method – assaying the time it took following a stop signal to halt movement towards the target. Specifically, we measured the time at which the cursor reached its maximum resultant displacement relative to the time of the stop signal (Fig. 2d), which we take to reflect the completion of the stop process. Overall, two kinematic estimates of SSRT were produced, one for failed stops of the home staircase (SSRT_K-H_) and another for successful stops of the target staircase (SSRT_K-T_).

Finally, we applied the BEESTS model separately to the home staircase and target staircase data (i.e., response times and probability of stopping as a function of the SSD) to estimate SSRT_B-H_ and SSRT_B-T_, respectively (see *BEESTS model* for details).

#### Variability of stopping latencies

The variability of SSRT was quantified in two ways. First, using the single-trial measurements of SSRT_K-H_ and SSRT_K-T_ (see Fig. 2 for example distributions) and calculating the intra-individual standard deviation for each (SD-SSRT_K__-__H_ and SD-SSRT_K-T_). Secondly, we applied the BEESTS model separately to the home and target staircase data to estimate the intra-individual standard deviation of SSRT (SSRT_B-H_ and SSRT_B-T_).

#### Trigger failures (TF)

TFs were quantified in two ways. First by assaying the proportion of stop trials (%) for the home staircase in which the cursor reached the target (TF_K-H_). Secondly, we applied the BEESTS model separately to the home and target staircase data (TF_B-H_ and TF_B-T_).

#### BEESTS model

We ran the BEESTS model (Matzke et al., 2017) using the Dynamic Models of Choice software (Heathcote et al., 2019) written in R Studio (1.1.463). The methods closely follow those used in the paper by Skippen et al. (2019), except that we applied the two-runner model which assumes a race between a go and stop runner, rather than the three-runner model (stop, correct go and go choice error), because choice errors were extremely rare (Table 3). Briefly, BEESTS estimates the distribution of the SSRT by using the participant’s go response time distribution, and by considering the failed stop response times as a truncated go response time distribution. The truncation points are randomly sampled from the SSRT distribution on each stop trial. The response time distribution underlying the go and stop processes is assumed to have both a Gaussian and exponential component and is described by three parameters (μGo, σGo, τGo and μStop, σStop, τStop). For such ex-Gaussian distributions, the mean and variance of the response time distributions are determined as μ + τ and *σ*^2 + τ^2, respectively. The model also estimates the probability of trigger failures for each participant, which are first projected from the probability scale to the real line with a probit transformation (standard normal cumulative distribution function).

**Table 3:** Performance in the stop signal task across experiments 1 and 2 as measured by the Race Model and kinematic method

Variable	Variable label	Staircase	Exp. 1 (*n* = 23)	Exp. 2 (*n* = 34)
Basic behavioral performance				
Go errors (%)	Omissions		0.1 ± 0.3	0.3 ± 0.5
	Choice errors		0 ± 0	0.4 ± 0.8
	Missed target		4.5 ± 3.5	3.9 ± 3.3
	False alarms		1.6 ± 1.2	1.5 ± 2.0
Go response latency (ms)	Reaction time		424 ± 53	468 ± 85
	Total response time		836 ± 83	931 ± 122
Failed stop response latency (ms)	Reaction time	Home	397 ± 49	444 ± 77
	Total response time	Target	768 ± 83	857 ± 113
Stop signal delay (ms)	SSD	Home	103 ± 55	157 ± 109
	SSD	Target	500 ± 76	633 ± 134
Stop success (%)	pStop	Home	45 ± 6	45 ±6
	pStop	Target	49 ± 3	50 ± 3
Race Model analysis				
Stopping latency (ms)	SSRT_RM_	Home	337 ± 54	329 ± 59
	SSRT_RM_	Target	338 ± 85	297 ± 65
Kinematic analysis				
Stopping latency (ms)	SSRT_K_ (ms)	Home	329 ± 63	327 ±56
	SSRT_K_ (ms)	Target	334 ± 89	331 ±53
Variability of stopping latency (ms)	SD-SSRT_K_ (ms)	Home	57 ± 19	64 ±25
	SD-SSRT_K_ (ms)	Target	56 ± 22	67 ±27
Trigger failures (%)	TF_K_	Home	0.9 ±1.3	1.4 ±3.5

Key: *pStop* probability of stopping, *SSD* stop signal delay, *SSRT* stop signal reaction time, *SD-SSRT* standard deviation of stop signal reaction time, *TF* trigger failures

We used the Bayesian parametric method (BPE) to estimate the parameters of the distributions, where the group-level mean and standard deviation parameters describe the group-level distribution for each parameter, and individual subject parameters are modeled using the group-level distributions. This approach is thought to be more accurate than fitting individual participants and is effective when there is less data per participant (Matzke et al., 2013, 2017). Participants’ behavioral data were initially modelled separately using a weakly informative set of uniform priors, and these fits were then used to set the population level mean prior in a second run that focused on the hierarchical fit. Specifically, for the initial uniform priors, the parameter μ for both the Go and the Stop process was truncated at 0 and 2000 ms, and for the σ and τ parameters it was 0 and 1000 ms. The prior for the trigger failure parameter was truncated at –6 and 6, as this would cover the entire range of the distribution. For the hierarchical fitting, we used normal hyper-prior distributions for the population-mean parameters. The truncation points were kept the same as in the uniform case, but with the difference that for the trigger failure parameter, the truncation was set at -Inf and Inf. Group-level standard deviations for the hierarchical fit were set to 1 for all parameters. The code with priors and truncation points can be found at (https://osf.io/d6a92/). The posterior distributions for each parameter were estimated using the Markov chain Monte Carlo (MCMC) sampling and the procedure followed that of Skippen et al. (2019). Participants were first modeled separately until the MCMC chains converged, as indicated by a Gelman-Rubin (R̂) statistic R̂<1.1. Participant fits then informed the start values for the hierarchical fit. We assessed the goodness-of-fit of the using posterior predictive model checks, which involves randomly selecting a set of parameter vectors from the joint posterior of the participant-level model parameters and using this to generate sets of stop signal data. We then visually assessed whether the predicted data closely resembled the observed data, focusing on the go and failed stop response time distributions, inhibition functions (pStop as a function of SSD), failed stop response times as a function of SSD.

We report the mean and 95% confidence interval of the population level mean parameters, focusing on the SSRT [μ + τ], SD-SSRT [√(*σ*^2 + τ^2)] and TF to enable comparisons at the group level with the same estimates produced by the Race Model and kinematic methods.

#### Number of trials required to evaluate stopping performance from movement kinematics

In order to investigate how many trials are required to obtain reliable measures of stopping performance with our kinematic approach, we ran post hoc simulations on the data. We randomly sampled *x* number of trials (from 5 to 70 in increments of 5) from each performance metric (SSRT_K_, SD-SSRT_K_ and TF_K_). Under the assumption that our summary average for each individual reflected the ‘true’ value, we asked how many trials would be required to obtain values within an arbitrary ± 5 % of this true value. We ran the simulation 1000 times at each increment of *x* trials.

#### Data and code

We provide the current data set and Matlab code used to perform the main behavioral analyses on the Open Science Framework (https://osf.io/d6a92/ ). The code used to run the tasks is also available.

#### Statistical analyses

Analyses were performed in Matlab 2021a (The Mathworks Ltd.). Most data are reported as mean ± standard deviation and were analyzed using paired *t* tests and linear regression (statistical significance accepted at *p* < 0.05). However, for output from the BEESTS model we report the posterior mean of the group-level data and the associated 95% credible intervals. Instead of using *t* tests when contrasting estimates from BEESTS with those produced by the other methods, we evaluated whether or not our Race Model and kinematic estimates fell within the credible intervals associated with the posterior distributions for each variable. This was to account for the fact that there is uncertainty in point estimates from the model (Matzke et al., 2017) that could unduly affect comparisons when using the frequentist *t* tests. We were still interested comparing estimates at the individual level, and hence did still apply linear regression analyses to the individual participant parameters, acknowledging again that there is uncertainty in these point estimates and that the correlations reported may be ‘over-confident’ (Skippen et al., 2019). Bayes Factors (BF_10_) were computed for *t* tests and correlations, and were interpreted as follows: 1/10-1/3, substantial evidence for the null hypothesis (H_0_); 1/3-1, anecdotal evidence for H_0_; 1-3, anecdotal evidence for the alternative hypothesis (H_1_); 3-10, substantial evidence for H_1_; 10-30, strong evidence for H_1_; 30-100, very strong evidence for H_1_; and >100, extreme evidence for H_1_. Response times and single-trial SSRT_K_ estimates were considered outliers and removed if they exceeded 1.5 times the inter-quartile range of the first and third quartiles, or if they were <100 ms.

## Results

Overall, the results from experiments 1 and 2 were very similar, and therefore we discuss them simultaneously.

### Basic go performance

Performance on the primary go task was good, with overall very few omissions, missed targets, false alarms, and choice errors (Table 3). As expected, reaction times and total response time differed, with the latter being approximately double the former (Table 3).

### Basic stop performance

Although our primary aim here was to develop direct, single-trial measurements of stopping latency and trigger failures using movement kinematics, we begin by presenting the results of the Race Model analyses. The Race Model forms the backbone of most stopping research but is rarely used to study reaching movements or the stopping of ongoing, as opposed to planned, movements [though see (Atsma et al., 2018; de Jong et al., 1990; Morein-Zamir et al., 2004; Venkataramani et al., 2018)].

The staircasing procedure for the stopping aspect of the task worked well. The different stop performance criteria for the two staircases led to an expected greater SSD for the target versus home staircase (experiment 1, *t* = 24.5, *p* < 0.001, BF_10_ > 100; experiment 2, *t* = 23.7, *p* < 0.001, BF_10_ > 100). pStop was close to 50% for both staircases (Table 3), although for some participants with fast go reaction times, the home staircase SSD hit the floor (set to 33.3 ms) meaning that pStop was slightly lower than 50%. This led to a small difference in pStop for the home compared to the target staircase (experiment 1, *t* = 2.7, *p* = 0.0127, BF_10_ = 9; experiment 2, *t* = 4.5, *p* < 0.001, BF_10_ > 100). Stopping latency estimates derived via the Race Model (Table 3) seemed broadly sensible given those in the literature utilizing button presses [~200–320 ms; (Aron et al., 2007; Hannah et al., 2020; Jana et al., 2020; Skippen et al., 2019; Smittenaar et al., 2013; van den Wildenberg et al., 2009; Weigard et al., 2019)]. Importantly, Race Model-derived SSRT estimates for the home and target staircases were similar at the group level in experiment 1 (*t* = 0.03, *p* = 0.98, BF10 = 0.9; Table 3), though there was a small difference between them in experiment 2 (*t* = 3.61, *p* < 0.001, BF10 = 90.7; Table 3). The estimates were nevertheless correlated with one another at the individual level (experiment 1, *r* = 0.43, *p* = 0.042, BF_10_ = 1.24; experiment 2, *r* = 0.59, *p* < 0.001, BF_10_ > 100). This implies that the stopping of planned and ongoing actions relies on a common process, an idea that is strongly supported by the very high correlations between home staircase Race Model and kinematic estimates that we come to shortly.

**Fig. 3:**
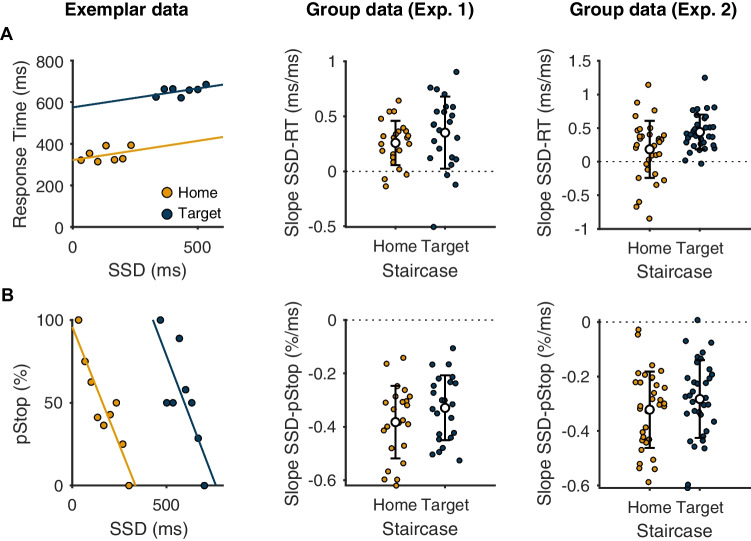
Exemplar data from single participants and group-level data showing that standard behavioral performance from both home and target staircases conform to various predictions of the Race Model, and that stopping performance was similar in the planning (home staircase) and execution (target staircase) stages of a movement. **a** Failed stop response times increase with the stop signal delay (SSD), as indicated by a positive regression slope. **b** The slope of the inhibition functions was similar across home and target staircases, indicative of similar stopping performance across the two staircases. Exemplar data in A and B have been averaged across trials for each SSD for the sake of visualization, but linear regressions were performed on single-trial data

In order to provide further evidence that the stopping of planned and ongoing reaching movements rely on a common process explainable by the Race Model, we interrogated how performance in the task varied as a function of SSD. First, we analyzed the single-trial relationship between failed stop response times and SSD. In line with predictions of the Race Model that longer SSDs allow longer response times to escape inhibition (Logan & Cowan, 1984), we found that failed stop response times increased as a function of SSD (as indicated by a positive slope, Fig. 3a). Second, we examined the relationship between pStop and SSD (i.e., the inhibition function) and found the slopes to be statistically similar for the home and target staircases (Fig. 3b). This result tentatively supports the idea of a similar stop process being engaged in each case, though we acknowledge that the inhibition function can be influenced by factors other than the efficacy of the stop process [e.g., presence of trigger failures; (Band et al., 2003; Matzke et al., 2017)].

### Direct measurement of stopping performance

Having shown that the data conform to various predictions of the Race Model, we reasoned that we could directly quantify stopping latency by examining the movement kinematics and quantifying the time at which ongoing movements are cancelled.

#### Home staircase: stopping during the early stage of movement execution

Here we assayed the stopping of ongoing movements shortly after their initiation, and therefore close in time to the planning stage evaluated when applying the Race Model to the same staircase. Despite the trials of interest being classified as failed stops according to the home staircase criterion, individuals still stopped before reaching the target in nearly every trial (experiment 1, 99.1 ±1.3 % of stop trials; experiment 2, 98.5 ±3.6 % of stop trials; also see Figs. 1c and 2a). This was also evident in the fact that movement amplitudes in failed stop trials were considerably smaller compared those in go trials (experiment 1: 130 ±97 pixels vs. 431 ±90 pixels, t = 14.0, *p* < 0.001, BF_10_ > 100; experiment 2: 97 ±86 pixels vs. 417 ±73 pixels, *t* = 18.4, *p* < 0.001, BF_10_ > 100), where amplitudes were measured as the maximum resultant displacement of the cursor relative to the point at which the cursor left the home pad.

**Fig. 4:**
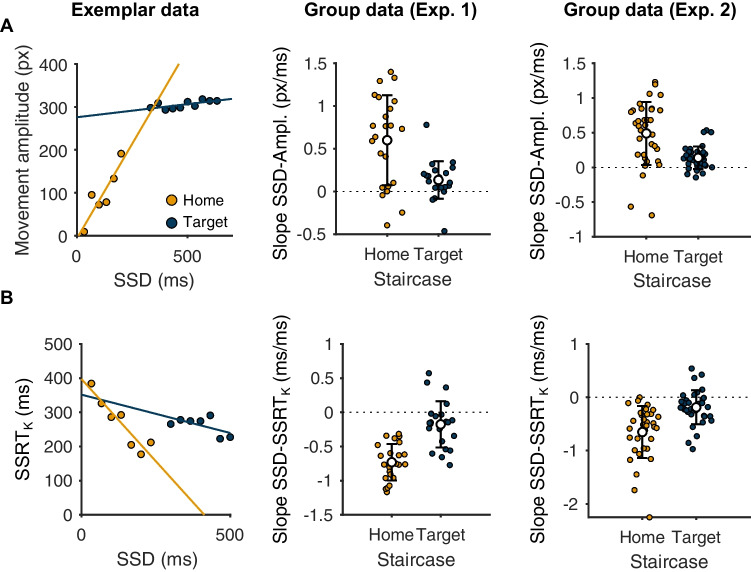
Exemplar data from a single-participant and group-level data showing behavior on trials where movements are initiated, but stopped before the target, follow predictions of the Race Model for the home and target staircases. **a** Movement amplitudes increase as a function of stop signal delay, as indicated by a positive regression slope. **b** Kinematic estimates of SSRT decrease as a function of stop signal delay, as indicated by a negative regression slope. Exemplar data in A and B have been averaged across trials for each SSD for the sake of visualization, but linear regressions were performed on single-trial data

As a further step before interrogating single-trial stopping latencies, we analyzed the single-trial level relationship between peak movement amplitudes and SSD for trials where the movement was initiated but was stopped before the target. Movement amplitudes tended to increase with SSD (Fig. 4a), and this was true for both staircases. This is again consistent with the Race Model, since longer SSDs permit the go process to be active for a longer period of time and so for the movement to progress further before eventually being interrupted (Atsma et al., 2018; Coxon et al., 2006; Jana et al., 2020). Linear regression also indicated that SSRT_K_ tended to decline as a function of SSD (Fig. 4b), in line with the Race Model prediction that at long SSDs only the fastest stop processes were quick enough to win the race.

**Fig. 5:**
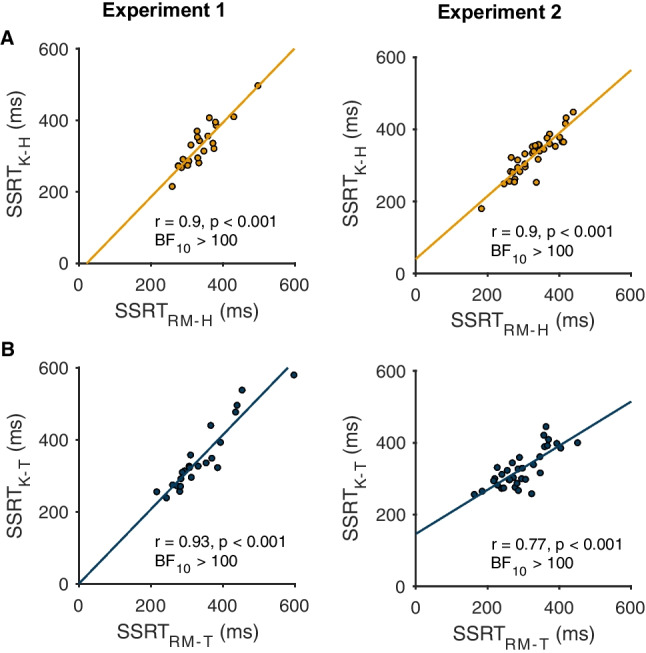
Relationships between Race Model and kinematic estimates of SSRT for home (**a**) and target (**b**) staircases across both experiments

**Fig. 6:**
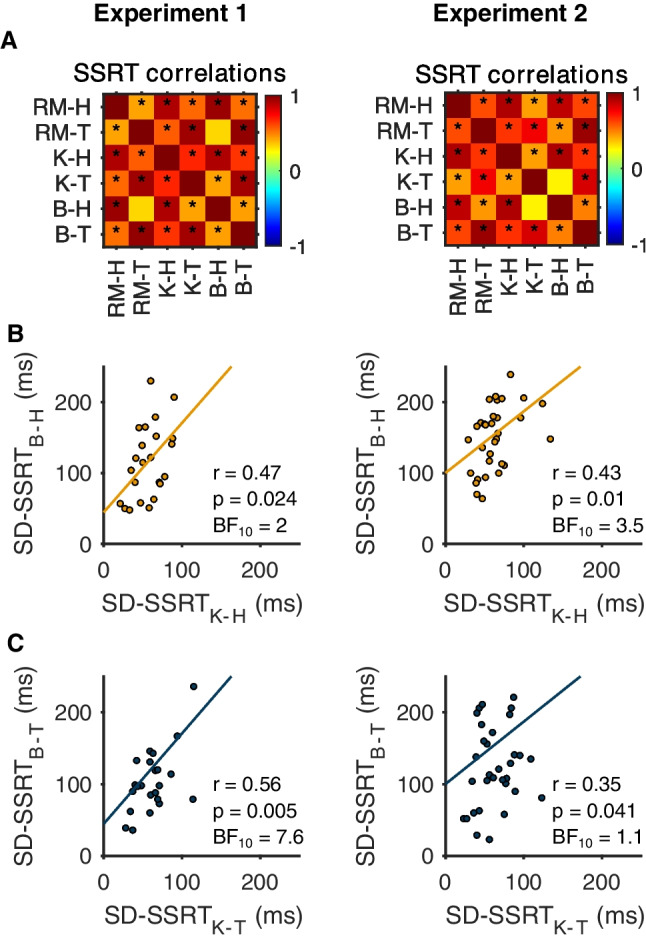
Correlations across estimates of stopping behavior derived from BEESTS (B), movement kinematics (K) and the Race Model (RM) for experiments 1 and 2. **a** Correlation matrix across different estimates of stop signal reaction time (SSRT) for the home (H) and target (T) staircases. BEESTs estimates of SSRT were positively related to Race Model and kinematic estimates in both experiments. ‘*’ indicates significant correlation. **b** Intra-individual standard deviation of SSRT estimates derived from movement kinematics (SD-SSRT_K_) were positively related to BEESTS estimates (SD-SSRT_B_) for the home staircase. **c** Intra-individual standard deviation of SSRT estimates derived from movement kinematics (SD-SSRT_K_) were positively related to BEESTS estimates (SD-SSRT_B_) for the target staircase

We then quantified stopping latencies on those same ‘failed stop’ trials of the home staircase. Kinematic estimates of SSRT were comparable with Race Model estimates (*t* = 1.52, *p* = 0.14, BF_10_ = 1.6, mean difference 9 ±28 ms; experiment 2, *t* = 0.29, *p* = 0.77, BF_10_ = 0.8, mean difference 1 ±32 ms; Table 3), and the two were very highly correlated with another in both experiments (Fig. 5a and Fig. 6a). This convincingly shows that stopping during the planning stages (SSRT_RM_) of movement and during the early stages of movement execution (SSRT_K-H_) relies on heavily overlapping processes. Our kinematics-based measurement therefore appears to provide sensible readouts of the latency of stopping and does so on a single-trial basis. This is useful, because it therefore allows a direct estimate of the intra-individual variability in the latency of stopping across trials, which was ~60 ms in both experiments (Table 3 and see Fig. 6b).

It was notable that some participants did, on occasion, still fail to stop before the target in home staircase trials (~1% stop trials at the group level; see Fig. 2a for an example). Less than half the sample exhibited this type of failure (10/23 individuals in experiment 1 and 12/34 in experiment 2). These outright failures occurred despite the fact participants had plenty of time to stop. For example, taking only those people exhibiting this type of stop failure in experiment 1, the mean stop signal delay for these trials was 192 ms and mean total response time was 759 ms, meaning any stop process had 576 ms on average to intervene. This is nearly twice the mean SSRT of these individuals (~330 ms). Moreover, if one considers the distribution of stopping times, the time available to stop exceeded the mean plus 3 intra-individual standard deviations of the SSRT. So even the very slowest stop processes had time to intervene. The simplest interpretation, therefore, is that these 1% trials represent those where the stop process was simply not triggered, i.e., trigger failures.

This estimate of trigger failures could be considered conservative, since it only looks at extreme cases where the participant entirely failed to stop. One could imagine that the relatively long response window and non-ballistic nature of the movement might permit the use of slower stopping mechanisms in cases where faster stopping mechanisms failed to be triggered, and hence some trials that would otherwise be registered as trigger failures were instead successfully stopped. This could include the use of slower neuroanatomical pathways that have been implicated in stopping under certain circumstances (Jahfari et al., 2012; Leunissen et al., 2016; Majid et al., 2012, 2013; Zandbelt et al., 2013). Alternatively, it is possible that on some occasions, the individual initially failed to trigger the stop process, resulting in the initiation of movement, but upon detecting the error (i.e., the failure to stop the initiation of movement) rapidly triggered a stop process and cancelled the movement before it reached the target. We think this is unlikely because the error must first occur, be detected, and then trigger/implement the stop process, yet the average time from movement onset to movement end was only ~150 ms for these failed stop trials. This seems too quick for an error-driven stop process to cancel the movement, particularly given that SSRT was typically >300 ms. Moreover, participants were not aware of the criterion for the home staircase, wherein leaving the home pad is classed as a failure to stop, and so the initiation of the movement would not have been perceived as an error.

#### Target staircase: stopping during the late stage of movement execution

The pattern of results here was very similar to that of the home staircase. As expected, movement amplitudes were smaller for successful stops of the target staircase compared to go trials (experiment 1: 393 ±86 pixels vs. 431 ±90 pixels, *t* = 12.5, *p* < 0.001, BF_10_ > 100; experiment 2: 387 ±71 pixels vs. 403 ±86 pixels, *t* = 13.0, *p* < 0.001, BF_10_ > 100). Movement amplitudes tended to increase as a function of SSD too, consistent with predictions of the Race Model (Fig. 4). At the group level, kinematic estimates of SSRT were highly consistent with Race Model estimates in experiment 1 (*t* = 1.67, *p* = 0.11, BF_10_ = 1.8, mean difference –12 ±34 ms) but differed slightly in experiment 2 (*t* = 15.9, *p* < 0.001, BF_10_ > 100, mean difference 32 ±42 ms; Table 3). The two estimates were very highly correlated across individuals in both experiments (Fig. 5b and Fig. 6a). SD-SSRT was ~60 ms and comparable to home staircase estimates (Table 3 and see Fig. 6c).

### BEESTS

We applied the BEESTS model to the data in order to compare our direct estimates of stopping latency and its variability, as well as the proportion of trigger failures. The Gelman-Rubin R̂ statistic was below the recommended criterion (i.e., <1.1) for each parameter in each data set (home and target staircases of experiments 1 and 2), and convergence was further confirmed by visual inspection of the MCMC chains. Overall, the data were well fit by BEESTS at the group level, as indicated by the posterior predictive data matching the observed inhibition functions, go trial total response time and failed stop reaction time distributions, and the relationship between failed stop response times and the SSD for each staircase in each experiment (see https://osf.io/d6a92/). BEESTS produced similar SSRTs to those of the other methods (see Tables 3 and 4), as indicated by the group-level 95% credible intervals containing the estimates provided by Race Model and kinematic method. The only exception was Target staircase in experiment 2, where the kinematic estimate fell just outside the credible interval (by ~20 ms).

**Table 4:** Performance in the stop signal task across experiments 1 and 2 as indicated via BEESTS. Data are posterior mean and the 95% credible intervals of the group-level mean parameters

Variable	Variable label	Staircase	Exp. 1 (*n* = 23)			Exp. 2 (*n* = 34)		
			Posterior mean	Credible interval		Posterior mean	Credible interval	
				2.5%	97.5%		2.5%	97.5%
Go response latency (ms)	RT		407	356	461	467	414	508
	TRT		815	752	888	923	850	984
Stopping latency (ms)	SSRT_B_ (ms)	Home	305	242	378	315	219	379
	SSRT_B_ (ms)	Target	291	219	377	253	208	309
Variability of stopping latency (ms)	SD-SSRT_B_ (ms)	Home	53	3	124	105	17	171
	SD-SSRT_B_ (ms)	Target	55	3	119	57	3	132
Trigger failures (%)	TF_B_ (%)	Home	0	0	0.009	0	0	0.005
	TF_B_ (%)	Target	0	0	0.004	0	0	0.004

Key: *pStop* probability of stopping, *RT* reaction time, *SSRT* stop signal reaction time, *SD-SSRT* standard deviation of stop signal reaction time, *TF* trigger failures, *TRT* total response time

The general consistency across methods also held for the standard deviation of SSRT, where kinematic estimates fell within the credible intervals indicated by BEESTS (Table 3 and Table 4). However, the mean and 95% credible interval for BEESTS-estimated trigger failures were so small as to all be essentially zero (Table 4), suggesting trigger failures contributed little to the model. This meant that the credible intervals did not contain the kinematic estimate of trigger failures (Table 3). Nevertheless, the results are still compatible overall because the kinematic measure also indicated there were very few trigger failures at the group and individual level.

We were also interested in comparing each variable at the level of individual participants. Overall, posterior predictive checks indicated that the model provided a reasonable representation of the data for most individual participants. BEESTS estimates of SSRT correlated very highly with the Race Model and kinematic estimates (Fig. 6a), and the mean of the individual estimates were also similar overall (experiment 1: home, 343 ±65 ms; target, 331 ±88 ms; experiment 2: home, 342 ±62 ms; target 289 ± 53 ms; compare with Table 3). BEESTS estimates of the standard deviation of stopping latencies were moderately related to kinematic estimates for the home and target staircases, despite the fact that the former tended to exceed the latter at the individual level (Fig. 6b and c). Since BEESTS-derived trigger failures were essentially 0 for all participants we did not attempt to correlate these values with the kinematic estimates.

### Number of trials required to obtain precise estimates of stop performance

The total number of stop trials required to obtain a precise estimate of SSRT_K_, SD-SSRT_K_ and TF_K_ was ~27, ~58 and ~29 (Table 5). Given a task with 25% stop signals, as in the present study, one would need ~100 total trials for SSRT_K_ and TF_K_ (the latter derived only from those people showing at least one TF), and ~240 trials for SD-SSRT_K_.

**Table 5:** Estimated total number of stop trials within a given staircase required to achieve an estimate of stopping performance within ±5% of the mean

	Experiment 1		Experiment 2	
	Home	Target	Home	Target
SSRT_K_	25 ± 10	26 ± 10	26 ± 13	30 ± 13
SD-SSRT_K_	59 ± 16	56 ± 7	59 ± 12	57 ± 8
TF_K_	28 ± 3	-	30 ± 4	-

## Discussion

We developed a version of the stop signal task that required participants to respond by making reaching movements with a computer mouse and used movement kinematics to provide single-trial readouts of key performance metrics. Despite using a different mode of responding (reaching movements) to standard tasks and assaying the stopping of ongoing (as well as planned) movements, we found that behavior in the task was well explained by the standard Race Model and BEESTS model. Across the two experiments, we found that kinematically derived estimates of SSRT, SD-SSRT, and TF corresponded well with model estimates at the group and individual level, particularly SSRT which showed very strong correlations across the different methods. Overall, we conclude that our approach has good face and convergent validity and offers a range of practical benefits over computational methods of assaying action-stopping.

### Stopping in the current task versus standard versions of the stop signal task

The data were well described by both the Race Model and BEESTS, suggesting that stopping in our task and standard versions of the task share common underlying processes and principles. This is important because both models have typically been employed to study the stopping of planned saccades or finger movements [e.g. (Hanes et al., 1998; Logan & Cowan, 1984; Matzke et al., 2013, 2017)], whereas we applied the models to a task examining the stopping of whole-arm reaching movements in two distinct phases.

The notion that common principles and processes underlie the stopping of different actions is supported by previous studies that have successfully applied the Race Model to the stopping of speech production (Xue et al., 2008), hand grip (de Jong et al., 1990), wrist (Brunamonti et al., 2012) and elbow flexion/extension (McGarry & Franks, 1997), and whole arm reaching/pointing (Atsma et al., 2018; Mirabella et al., 2009; Venkataramani et al., 2018). Indeed, one study showed that the stopping of finger, wrist and whole-arm movements was well explained by the Race Model, and that SSRT estimates and inhibition functions were similar across movements within the same individuals (Brunamonti et al., 2012).

Another line of evidence comes from neuroscience studies in humans and primates which suggest that the stopping of eye (Gulberti et al., 2014; Isoda & Hikosaka, 2008; Jarvstad & Gilchrist, 2019; Wessel et al., 2013), hand (Aron & Poldrack, 2006; Bastin et al., 2014; Ghahremani et al., 2018; van den Wildenberg et al., 2006; Wagner et al., 2018), speech effectors (Cai et al., 2012; Ghahremani et al., 2018; Wagner et al., 2018; Xue et al., 2008) and whole-arm reaching (Mirabella et al., 2012; Pasquereau & Turner, 2017) share some of the same prefrontal-basal ganglia circuitry and neurophysiology [see (Hannah & Aron, 2021; Wessel & Aron, 2017) for reviews].

Our data are also consistent with the idea that (non-ballistic) movements can be interrupted during the planning and execution phases [see Fig. 1a and Fig. 2; (Atsma et al., 2018; de Jong et al., 1990; Georgopoulos et al., 1981; Kudo & Ohtsuki, 1998; McGarry & Franks, 1997; Venkataramani et al., 2018)] and we presume that the underlying processes may be similar in each case. This is suggested by the fact that SSRT measured during the planning phase (SSRT_RM-H_) is highly compatible with SSRT measured during the execution phase (e.g., SSRT_RM-T_ and SSRT_K-H_) [and see (Morein-Zamir et al., 2004)]. Computational modelling of stopping planned and ongoing reaching movements corroborates this view (Venkataramani et al., 2018). Finally, neurophysiological studies, using two distinct markers of cortical inhibitory processing, have shown that the stop process proceeds in the same way regardless of how long the go process has been active for (de Jong et al., 1990; Jana et al., 2020). Taken together, we suppose that stopping in the planning and execution phases of movement can be understood in terms of a common set of underlying processes, as described by the Race Model, and that our kinematic methods are applicable to the wider study of action-stopping.

### SSRT

Our Race Model-based estimates of SSRT (~330 ms) were broadly consistent with that of previous work utilizing continuous measurement of movement kinetics/kinematics to directly quantify SSRT [~300 ms (Morein-Zamir et al., 2004, 2006; Schultz et al., 2021)], as well as studies applying the Race Model to the stopping of button press responses [200-320 ms; (Aron et al., 2007; Hannah et al., 2020; Jana et al., 2020; Skippen et al., 2019; Smittenaar et al., 2013; van den Wildenberg et al., 2009; Weigard et al., 2019)] and whole-arm movements [200–250 ms (Atsma et al., 2018; Brunamonti et al., 2012; Mirabella et al., 2009)]. We suspect the fact that our estimate is at the upper end of the range within previous literature could be related to differences in the task requirements across studies (e.g., the use of different muscles/joints and apparatus that lend to differences in inertia when making or cancelling a response), along with differences in the criteria used to define a successful stop.

As has already been mentioned, there was a high level of agreement between the kinematic estimates of SSRT and those produced by the Race Model at both the group and individual level. They also closely matched estimates provided by the BEESTS model. This triangulation of SSRT estimates provides strong support for the convergent validity of our kinematic method and is reassuring given the different potential sources of bias in each of the measures. For example, kinematic estimates of SSRT are potentially biased by the fact that for the home staircase, SSRT_K_ was measured from failed stop trials, whereas for the target staircase it was measured from successful stop trials. By implication, SSRT_K_ for the home staircase will be biased towards trials where the stop process was slower (more fast stop processes are excluded from the distribution), and the opposite is true for the target staircase. Additionally, the Race Model only provides a good approximation of ‘true’ SSRT when model assumptions hold, SSRT is constant, go RTs are not heavily skewed, and the trigger failure rate is zero (Band et al., 2003; Matzke et al., 2013). It seems that any such biases in SSRT estimation in the current data were small, but this may not be true in all data sets. Therefore, it may be generally advisable to use BEESTS alongside the kinematic method to assess the degree of bias in stopping distributions as an initial step before making further inferences regarding kinematic estimates of stopping performance.

### Standard deviation of SSRT

We were able to read out stopping latencies at the single-trial level, and this in turn allowed us to directly quantify intra-individual standard deviation of stopping latencies as ~60 ms. This value approximates those in a previous study using continuous kinetic measurements to read out SSRT at the single-trial level [SD-SSRT ~50 ms (Morein-Zamir et al., 2006)], as well a previous electromyography-based estimates [SD-SSRT ~35–45 ms (Goonetilleke et al., 2010; Jana et al., 2020)]. A strength of the present work is that we contrasted our direct measurement approach with a computational method to examine the degree of convergence between them.

We found that BEESTS provided standard deviation values comparable with those of our kinematic method at the group level, and moderately correlated at the individual level. Previous work had also shown a similar degree of correspondence between BEESTS estimates with those produced by single-trial electromyography-based measure of stopping latencies (Jana et al., 2020). Together, these findings support the convergent validity of both BEESTS and our kinematic approaches to quantifying the variability in stopping latencies.

### Trigger failures

The current trigger failure measure appears to possess good face validity. It represents those trials where there was seemingly sufficient time to stop and yet individuals did not stop, presumably because the stop process was not triggered. According to this measure, trigger failures occur very infrequently in the current stop signal task (~1% stop trials). In fact, most people did not have any trigger failures at all, meaning that most trigger failures at the group level were attributable to a sub-sample of individuals. We speculate that this heterogeneity may be of relevance to individual differences in real-world behavioral control, though this idea remains to be tested.

Although BEESTS estimates of trigger failures did not overlap with our kinematic estimate, the absolute values were in fact very close (~0 and ~1%). The reason for the lack of overlap can be explained by two factors. First, the task design afforded relatively few trigger failures overall, particularly compared to the 4-18% trigger failures indicated by BEESTS in the standard button pressing version of the task (Jana et al., 2020; Matzke et al., 2017; Skippen et al., 2019). Secondly, we employed the hierarchical fit which uses the group-level distributions to model the individual participant parameters, in order to make up for the fact that there were too few trials per person to ensure accurate fitting at the individual level (Matzke et al., 2013, 2017). The net result is that trigger failures contributed minimally to the group-level fits, and this then minimized the potential to fit them at the individual level. In other words, although such shrinkage is a desirable quality because it prevents over-fitting, in the present case, it combined with a very low prevalence of trigger failures meant that the model could not capture these subtle changes arising from small differences in the overall data distributions.

Why were trigger failures rare in our study? The answer does not seem to lie in the analysis methods, range of stop signal delays or features of the stop signal, as we found similarly low values across BEESTS vs. kinematics methods, home versus target staircases (with BEESTS) and different stop signals in experiment 1 and 2. Instead, it may be due to the long interval between responses (i.e. inter-trial interval and delay period), which allowed participants to move the mouse cursor back to the home pad and helped minimize the predictability of the go stimulus. The effect might have been that each trial began in a very deliberate and intentional manner. By comparison, typical button-press versions of the task have short inter-trial intervals meaning that responding becomes very repetitive and can be performed with less conscious attention to the go stimuli. This could then lead to a more general inattention to the task that extends to instances where the stop signal is presented and more effortful, top-down control is required, hence leading to a higher proportion of trigger failures. It is also possible that the absolute number of trigger failures is influenced by the response deadline. For example, a long response deadline might encourage long response times. This would afford more time for a stop signal to be detected and acted upon and could therefore reduce the probability of trigger failures. The converse would also be true in instances where the response deadline is very short. Although our aim here was simply to demonstrate that we could detect and quantify trigger failures, future work should seek to establish the influence of methodological parameters on the number of trigger failures. Increasing the overall propensity for trigger failures in the task would increase its utility as a tool for studying individual differences and single-trial neural correlates of trigger failures.

We note that our approach can only be used to estimate trigger failures at short stop signal delays (i.e., home staircase). We assume that the proportion of trigger failures remains constant across all delays, but this may not be true. BEESTS also makes this assumption but, with sufficient data quality could be used to test this assumption.

### Practical implications

The present method permits precise quantification of stopping performance indices with seemingly few trials: SSRT and TF can be estimated with ~100 trials in total. Admittedly, our approach to estimating measurement precision was somewhat crude, using an arbitrary 5% criterion, and does not speak to the accuracy of the estimates (though this is supported by the similarity with BEESTS estimates). As a point of reference, standard applications of the Race Model require at least 200 trials to produce reliable estimates (Verbruggen et al., 2019), and BEESTS requires 165–250 stop trials and hence >600 trials in total (Matzke et al., 2019, 2017).

A further benefit is that the current method does not require any special equipment to collect data, such as a strain gauge [e.g., (Morein-Zamir et al., 2006)] or EMG [e.g., (Atsma et al., 2018; Jana et al., 2020)], nor even a visit to the laboratory. Instead, it can be performed anywhere using a laptop or personal computer and a mouse. In fact, it was reassuring that results across the two experiments were highly replicable despite the many differences within and between them (e.g., populations, computer devices, local environment in which the tasks were performed and differences in go/stop cues). The task therefore has the potential for use in large-scale, cross-sectional studies examining the relationship between these metrics of behavioral stopping and real-world self-reports of behavioral control/impulsivity. Previous work showed that relationships between Race Model estimates of SSRT and real-world impulsivity/behavioral control are often moderate at best (Eisenberg et al., 2019; Friedman & Miyake, 2004; Lijffijt et al., 2004). Given the similarity between kinematic and Race Model estimates of SSRT, our method seems unlikely to improve this particular relationship, but the standard deviation and trigger failure measures may offer additional explanatory power in relation to real-world behavioral control. Further work is, however, required to examine the sensitivity of our approach to methodological issues, such as strategic response slowing (Verbruggen et al., 2013), before the task can be rolled out for large scale studies.

A potential downside of studying the stopping of ongoing movements is that biomechanical factors might play a bigger role in the absolute SSRT and SD-SSRT values than for the stopping of planned movements. For example, inertial movements might take some time to ‘brake’ and decelerate, and this could prolong the detection of the stop process in the movement kinematics. Approaches based on changes in acceleration might help to minimize any delay between the onset of the stop process at the level of the muscle and the kinematics. However, even these will be susceptible to factors such as the force-length and force-velocity profiles of the involved agonist-antagonist muscle pairs that undergo coordinated suppression/recruitment (Atsma et al., 2018; de Havas et al., 2020). Hence, trial-by-trial variations in the dynamics of the movement and inter-individual differences in biomechanical performance could add unwanted ‘noise’ or bias to estimates of SSRT intended to capture a cognitive process, rather than its biomechanical implementation. In practice, however, any influence on inter-individual variability appears small as SSRT estimates showed strong agreement across the execution and planning phases of movement when biomechanical factors were and were not involved, respectively.

Biomechanical confounds are also present in simple button pressing tasks (Jana et al., 2020). Consequently, some research has developed electromyography-based methods of measuring stopping latencies, which depend on the onset/offset of agonist/antagonist muscle activity (Atsma et al., 2018; Goonetilleke et al., 2010; Jana et al., 2020; Raud & Huster, 2017) and circumvent the inherent electromechanical delays and biomechanical factors involved in responding. Nevertheless, such methods also come with downsides. Some studies utilized intramuscular electromyography (Goonetilleke et al., 2010, 2012), an invasive tool that is not suited to all muscles or populations. Electromyographic markers also provide only a pseudo-single-trial measure of performance because the bursts of muscle activity used to quantify stopping latencies are only present in ~50 % of successful stop trials (Atsma et al., 2018; Jana et al., 2020). More generally, electromyographic methods require specialist equipment and are therefore not appropriate for online studies. Instead, they may be best suited to neuroscience studies focusing on the neural mechanisms of stopping.

## Conclusions

We introduced a novel version of the stop signal task that provides direct readouts of stopping behavior. In addition to the standard performance metric, SSRT, the task also provides estimates of the variability of SSRT and the proportion of trigger failures – measures that are not possible using the standard Race Model approach. These additional descriptors of stopping performance, along with the inter-individual heterogeneity they exhibit, makes this task a good vehicle for future individual differences studies into the psychology of behavioral control. It is possible that although SSRT correlates only poorly with self-reported behavioral control in the literature, the standard deviation of SSRT and trigger failures might fare better. Finally, our approach offers some advantages over the use of model-based estimations of stopping performance since it requires fewer trials to obtain a reliable estimate and offers single-trial readouts of performance.

## Abbreviations

BEESTS: Bayesian estimation of ex-Gaussian stop-signal reaction time
H: Home staircase
K: Movement kinematics
RM: Race Model
SSD: Stop signal delay
SSRT: Stop signal reaction time
SD-SSRT: Standard deviation of the stop signal reaction time
T: Target staircase
TF: Trigger failure
